# Synthesis and antimicrobial activity of new series of thiazoles, pyridines and pyrazoles based on coumarin moiety

**DOI:** 10.1038/s41598-023-36705-0

**Published:** 2023-06-19

**Authors:** Mariam T. Sayed, Salwa A. Elsharabasy, Anhar Abdel-Aziem

**Affiliations:** Chemistry Department, Faculty of Science (Girls), Al-Azhar University, Nasr City, 11754 Cairo Egypt

**Keywords:** Biochemistry, Drug discovery

## Abstract

Microbial infections are currently a widespread disease in hospitals and community health centres and are a major cause of death worldwide. In pursuit of searching new antimicrobial agents, coumarin linked to thiazoles, pyridines and pyrazoles have been developed and evaluated for their antimicrobial properties against two Gram + bacteria, two Gram − bacteria as well as two fungi. Some of the prepared coumarins displayed high to moderate activity against the tested microorganisms with respect to the reference drugs. However, compound **3** exhibited antimicrobial effect equal to the reference drug Ciprofloxacin for Gram − baceria *Enterobacter cloacae*. Compound **12** was found to be the most potent compound against *Bacillus pumilis* with MIC of 7.69 (µmol/ml). Compounds **3**,** 4** and **12** showed remarkable activity against *Streptococcus faecalis* with MIC of 14.34, 3.67 and 15.36 (µmol/ml), respectively. Regarding *Escherichia coli*, most compounds recorded high to moderate MIC values (4.73–45.46 µmol/ml). Moreover, in case of *E. cloacae* compound **9** was the most potent compound with MIC value of 22.76 (µmol/ml).

## Introduction

One of the main factor in lowering the worldwide burden of infectious illnesses is antimicrobial agents^1^. When bacteria, viruses, fungi, and parasites, among other microbes, are able to adapt and flourish in the presence of drugs, this phenomenon is known as antimicrobial resistance (AMR)^2^. Due to the unreasonable usage of antibiotics, the appearance of multidrug-resistant (MDR) pathogens increased resulting in greater mortality and morbidity. Methicillin-resistant Staphylococcus aureus (MRSA), vancomycin-resistant Enterococci (VRE), and MDR Gram-negative bacteria in particular cause numerous therapeutic medications to lose their effectiveness or completely stop working. Additionally, the highly invasive fungal infections have posed an unprecedented challenge to the health sector^3–5^. Depending on how they work, antimicrobial agents can be categorised into several classes. Inhibitors of protein synthesis, inhibitors of nucleic acid synthesis, inhibitors of metabolic processes, and agents that depolarize cell membranes are the primary categories^6,7^. Antibiotics can no longer be used to treat bacterial infections, indicating an uncertain future for healthcare. In order to combat medication resistance on clinically important infections, it is necessary to find novel compounds with antibacterial activity that may function through mechanisms of action that are different from those of well-known classes of antimicrobial drugs^8–11^.

Coumarins are heterocycles that are widely distributed in plants. Apricots, cherries, cinnamon, strawberries, and other foods are some excellent sources of coumarins^12^. Natural and synthetic coumarins demonstrated a broad range of therapeutic applications^13^, including antimicrobial^14–16^, anti-HIV^17,18^, antioxidant^19^, anticoagulant^20,21^, anti-infammatory^22,23^, anticonvulsant^24,25^, anticancer^26–28^ and antiviral^29^. Also, they attract the major interest of chemists due to their wide variety of uses such as laser dyes^30^, cosmetics^31^, fluorescence probes^32^, photosensitizers^33^, food and perfumes^34^. Additionally, they have proven to be novel lipid-lowering agents with mild triglyceride-lowering capability^35^.

Thiazole ring is present in a lot of commercial medications as an active component^36^ due to its marvelous biological activity such as antiviral^37^, antimicrobial^38,39^, anti-inflammatory^40^, anti-HIV^41^, antitumor^42,43^ and antioxidant. Moreover, thiazolyl–coumarin are a significant group of heterocycles having a wide range of biological functions such as antibacterial, anticancer, antiviral, antioxidant^44–48^. It is well known that coumarin derivatives with pyridine heterocycles have anticoagulant, antibacterial, antifungal properties and antiproliferative activity against cancer cell^49–52^. In this regard and in accordance with the data offered, we have been concentrating on the synthesis of molecular hybrids based on bioactive heterocycles and coumarins as well as the evaluation of their antimicrobial activity.

## Results and discussion

**Figure 1:**
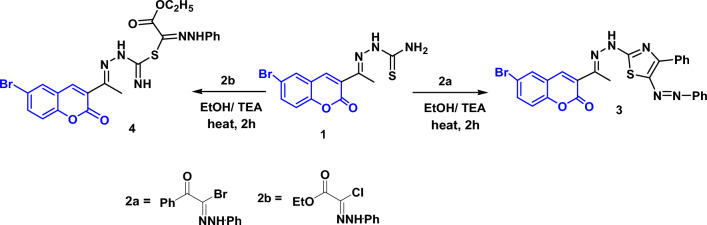
Reaction of compound **1** with hydrazonoyl halides.

A new series of thiazoles attached to 6-bromocoumarin moiety were synthesized by Hantzsch thiazole synthesis. Thus, interaction of 2-(1-(6-bromo-2-oxo-2*H*-chromen-3-yl)ethylidene)hydrazine-1-carbothioamide **(1)**^53^ with hydrazonoyl halides **2a, b** in ethanol and triethylamine under reflux gave the derivatives **3** and** 4**, respectively, in good yield (Fig. 1). The structures of the new derivatives were elucidated by elemental analysis and spectroscopic data.

The infrared spectra of compounds **3** and** 4** revealed the presence of stretching frequencies at 3429 and 3428 cm^−1^ attributable to the imino group, in addition to the presence of a strong absorption bands at 1735 and 1736 cm^−1^ due to carbonyl group as well as the other absorption bands. Moreover ^1^H NMR spectrum in DMSO-*d*_6_ for compound **3** revealed singlet signals at chemical shifts 2.37 and 8.30 ppm for methyl and imino protons, respectively, beside other signals due to aromatic protons. Its ^13^C NMR (100 MHz) recorded signals at δ 17.90 (CH_3_), 116.82–159.43 (Ar–C) and 183.16 ppm (C=O). While in ^1^H NMR spectrum of compound **4 (**DMSO-*d*_6_), triplet and quartet signals were appeared at chemical shifts 1.31 and 4.37 ppm, besides other three singlet signals at chemical shifts 2.34, 7.23 and 8.59 ppm assignable to methyl and 2NH protons, respectively. The ^13^C NMR (100 MHz) of **4** displayed signals at δ 14.31, 17.89 (2CH_3_), 63.40 (CH_2_), 118.76, 118.94–159.11 (Ar–C), 166.28 and 195.45 ppm (2C=O).

**Figure 2:**
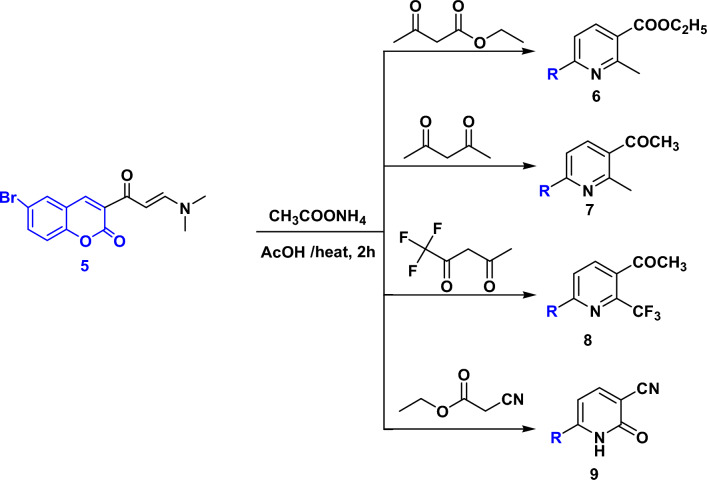
Synthesis of pyridines **6**–**9**.

Next, condensation of enaminone **5**^54^ with active methylene compounds such as ethyl acetoacetate, acetylacetone, trifloroacetylacetone and ethyl cyanoacetate in AcOH and AcONH_4_ resulted in the formation of new pyridines **6**–**9**, respectively as shown in Fig. 2. The structure of the new products was assigned based on the spectral data and elemental analysis. The IR spectra of pyridines **6**–**9** displayed two absorption bands at the region of 1675–1715 cm^−1^ and 1740–1710 cm^−1^ accounted for two carbonyl groups. ^1^H NMR spectrum for compound **6** as an example, in DMSO-*d*_6_ showed triplet and quartet signals at δ 1.34 and 4.27 ppm for (CH_3_CH_2_O-) and singlet signal at 2.90 ppm for methyl protons. In the ^13^C NMR spectrum of compound **6** two CH_3_ and one CH_2_ appeared at 14.35, 25.06 and 61.62 ppm, respectively. Signals at 158.76 and 166.21 ppm are attributed to two carbonyl groups. Whereas compound **7** recorded two singlet signals at δ 1.90 and 2.43 ppm for two methyl groups, beside four doublet signals at δ 6.80, 7.30, 7.70 and 8.40 ppm for six aromatic protons. In the ^13^C NMR spectrum of compound **7** two methyl groups appeared at 25.03 and 29.97 ppm, respectively. Signals at 168.38 and 170.41 are attributed to two carbonyl groups. Furthermore, compound **8** showed one singlet signal at δ 2.71 ppm assigned to COCH_3_ protons, all other signals were for CH aromatic protons. Compound **9** displayed a singlet signal at δ 7.95 ppm for NH proton as well as other signals for CH aromatic protons.

**Figure 3:**
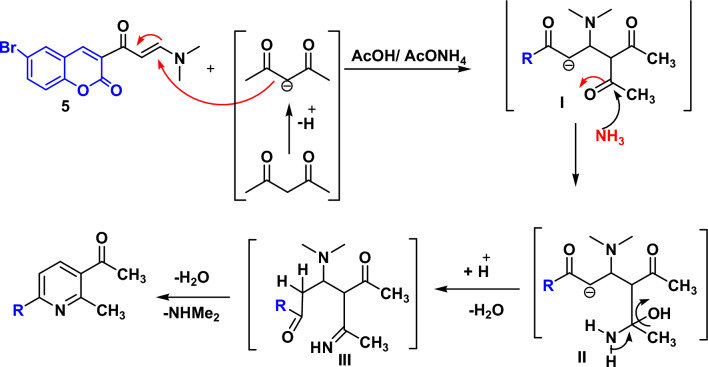
Mechanism of formation of pyridines.

The most likely pathway for the formation of pyridine derivatives **6**–**9** is outlined in Fig. 3. The reaction proceeded via initial Michael addition of the active methylene compound to the activated double bond of enaminone **5** to give I. Secondly**,** nucleophilic addition of NH_3_ molecule (generated from dissociation of ammonium acetate) to carbonyl carbon and subsequent cyclization via elimination of dimethyl amine and two water molecules lead to final product.

**Figure 4:**
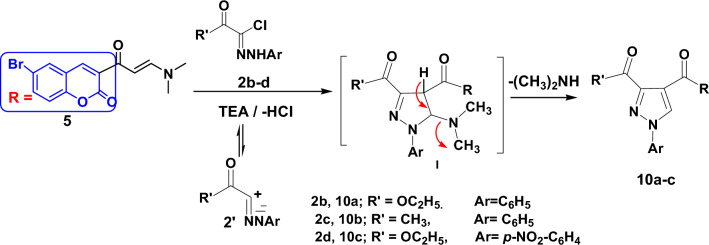
Synthesis of pyrazoles **10a–c**.

However, new pyrazoles **10a–c** were synthesized from the reaction of enaminone **5** and hydrazonyl halides **2b–d** in boiling benzene containing trimethylamine under reflux as outlined in Fig. 4. By using spectroscopic data and elemental analysis, all structures were clarified. ^1^H NMR spectrum of compound **10a** revealed new triplet and quartet signals for the ethoxy group at 1.16 and 4.20 ppm, while, pyrazole derivative **10b** recorded a singlet signal for the methyl protons at 2.57 ppm, beside the aromatic protons. The ^13^C NMR spectrum of compound **10b** showed one methyl groups at 25.8 ppm while three signals for carbonyl groups appeared at 146.10, 154.21 and 158.41 ppm, respectively. Also, compound **10c** recorded new triplet and quartet signals for the ethoxy group at δ 1.35 and 4.44 ppm in ^1^H NMR spectrum, its ^13^C NMR spectrum showed signals at 27.83 (CH_3_), 116.99–158.36 (Ar–C), 184.74, 194.51 (3C=O).

The mechanism^55^ for the formation of compounds **10a–c** is illustrated in Fig. 4. The reaction proceeded via 1,3-dipolar cycloaddition reaction between the imine **2′** (produced from the reaction of compound **2** with TEA) and the activated double bond in compound **5** giving the non-isolable cyclo adduct **I**. The intermediate **I** loses dimethylamine molecule to give the final pyrazole derivatives **10a–c**.

**Figure 5:**
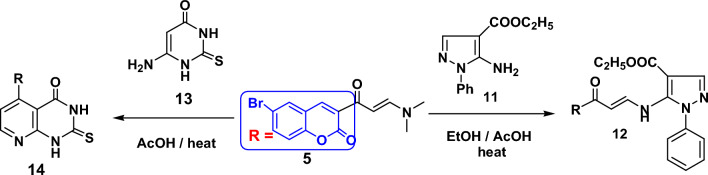
Reaction of enaminone with uracil and pyrazole.

On the next side, enaminone **5** was reacted with 5-Amino-1-phenyl-1*H*-pyrazole-4-carboxylic acid ethyl ester (**11**)^56^ in ethanol and drops of acetic acid to give 5-[3-(6-bromo-2-oxo-2*H*-chromen-3-yl)-3-oxo-propenylamino]-1-phenyl-1*H*-pyrazole-4-carboxylic acid ethyl ester (**12**) through elimination of dimethylamine molecule (Fig. 5). The IR spectrum of compound **12** revealed strong absorption peaks at ν 3265, 1703 and 1682 cm^−1^ for NH and two carbonyl groups respectively. Its ^1^H NMR spectrum recorded triplet and quartet signals at δ 1.2 and 4.19 ppm due to ethoxy group, beside a singlet signal due to NH proton at δ 8.59 ppm. In the ^13^C NMR spectrum of compound **12** one CH_3_ and one CH_2_ appeared at 14.93 and 45.02 ppm, respectively. Signals at 154.06, 158.76 and 164.03 are attributed to three carbonyl groups.

Finally, reaction of enaminone **5** with 6-aminothiouracil **13** in glacial acetic acid furnished 5-(6-bromo-2-oxo-2*H*-chromen-3-yl)-2-thioxo-2,3-dihydro-1*H*-pyrido[2,3-*d*]pyrimidin-4-one (**14**) in a good yield.

### Biological studies

#### Antimicrobial activity

The primary antimicrobial screening for the new compounds was assessed against six microbes using the agar well diffusion method. The chosen pathogenic microbes were *Bacillus pumilis* (MTCC-2296) and *Streptococcus faecalis* (MTCC-0459) as Gram + bacteria, *Escherichia coli* (ATCC-25955) and *Enterobacter cloacae* (ATCC-23355) as Gram – bacteria, while *Saccharomyces cerevisiae* (ATCC-9763) and *Candida albicans* (ATCC-10231) were used as fungi. Penicillin G was used as standard antibacterial (Gram +) drug while Ciprofloxacin was used as standard antibacterial (Gram −) drug and Ketoconazole as antifungal drug. The broth dilution method was performed to measure the minimum inhibitory concentration (MIC), minimum bactericidal concentration (MBC) and the minimum fungicidal concentrations (MFC).

Preliminary antimicrobial testing results (Table 1) demonstrated that thiazole derivative **3** having 5-phenyl azo group was high active against the two gram + bacteria with IZs of 20 and 20 mm and has the same IZ (23 mm) as Ciprofloxacin in the case of *E. cloacae*. While, compound **4** with phenyl hydrazo group attached to 5-position of thiazole nucleus revealed promising antibacterial effect against *E. coli* and *C. albicans* with IZs of 22 and 19 mm and was moderate active against *B. pumilis* (IZ = 18 mm). Regarding pyridine derivatives **6**, **8** and **9**, it was observed that compound **8** with CF_3_ group attached to 2-position of pyridine ring showed high activity towards one bacteria and one fungi comparing with compounds **6** and **9** with CH_3_ and C=O groups which was high active against one of tested bacteria. Potent antimicrobial effects were observed for compound **12** with ester group attached to 4-position of pyrazole ring towards all the tested pathogenic microbes (IZs = 19–21 mm) except with *C. albicans* whereas, pyrimidine thione **14** was high active only towards one bacteria and one fungi.

**Table 1:** In vitro preliminary antimicrobial activities of the new compounds against pathogenic bacteria and fungi.

Sample code	Inhibition zone diameter in mm					
	Gram (+) bacteria		Gram (−) bacteria		Fungi	
	*B. pumilis*	*S. faecalis*	*E. coli*	*E. cloacae*	*S. cerevisiae*	*C. albicans*
3	20	20	13	23	16	15
4	18	16	22	15	17	19
6	10	17	21	16	10	13
8	12	20	19	17	8	19
9	16	21	19	17	10	13
12	20	21	20	20	19	12
14	16	21	18	15	20	16
Penicillin G	25	24	–	–	–	–
Ciprofloxacin	–	–	29	23	–	–
Ketoconazole	–	–	–	–	22	23

#### MIC and MBC

The results for the minimum inhibitory concentration (MIC) and the minimum bactericidal concentration (MBC) (Table 2) indicated that compound **12** has the highest MIC of 7.69 (µmol/ml) for *B. pumilis* while other compounds revealed moderate MIC*.* Compounds **3**, **4** and **12** exhibited high MIC of 14.34, 3.67 and 15.36 (µmol/ml), respectively for *S. faecalis*. For *E. coli*, all compounds showed high MIC except compounds **4** and **9** which have moderate MIC of 29.41 and 45.46 (µmol/ml), respectively**.** Moreover, in case of *E. cloacae,* all compounds revealed moderate MIC except compound **9** which showed high MIC of 22.76 (µmol/ml). In case of the minimum bactericidal concentrations (MBC), all compounds exhibited moderate MBC for the two gram + bacteria. While, for gram – bacteria all compounds showed moderate MBC except compound **8** which has MBC of 37.84 (µmol/ml) for *E. coli*.

**Table 2:** In vitro MIC and MBC for the synthesized compounds.

Sample code	MIC (µmol/ml)				MBC (µmol/ml)			
	*B. pumilis*	*S. faecalis*	*E. coli*	*E. cloacae*	*B. pumilis*	*S. faecalis*	*E. coli*	*E. cloacae*
3	28.65	14.34	14.34	28.65	114.80	57.49	114.80	114.80
4	59.01	3.67	29.41	117.83	235.67	117.83	117.83	235.67
6	80.62	80.62	20.11	40.18	160.99	321.99	80.62	160.99
8	37.84	151.64	4.73	37.84	151.64	303.28	37.84	151.64
9	91.21	91.21	45.46	22.76	364.29	182.14	182.14	91.21
12	7.69	15.36	15.36	30.68	61.57	61.57	61.57	122.95
14	38.78	38.78	4.84	155.38	155.38	155.38	77.81	310.77
Penicillin G	0.37	11.69	–	–	1.49	46.68	–	–
Ciprofloxacin	–	–	0.48	0.75	–	–	1.50	3.01

#### MIC and MFC

The results for the minimum inhibitory concentrations (MIC) and the minimum fungicidal concentrations (MFC) were illustrated in Table 3. All compounds revealed moderate MIC values for both *S. cerevisiae* and *C. albicans* except compound **12** which showed the highest MIC of 15.36 (µmol/ml) for *S. cerevisiae.* On the other hand, all compounds exhibited moderate MFC for the two fungi except compound **3** which has the lowest MFC of 459.2 (µmol/ml).

**Table 3:** In vitro MIC and MFC of the newly synthesized compounds.

Sample code	MIC (µmol/ml)		MFC (µmol/ml)	
	*S. cerevisiae*	*C. albicans*	*S. cerevisiae*	*C. albicans*
3	28.65	114.80	114.80	459.20
4	117.83	29.41	235.67	117.83
6	80.62	80.62	321.99	321.99
8	151.64	75.94	303.28	151.64
9	182.14	45.46	364.29	182.14
12	15.36	61.57	61.57	122.95
14	155.38	155.38	310.77	310.77
Ketoconazole	0.94	0.23	3.76	0.94

## Experimental protocols

### General informations

All reagents and solvents were of commercial grade. Melting points of all the prepared coumarins were detected on an electro thermal apparatus and may be uncorrected. IR spectra were recorded using the Nicolet is 10 FTIR instrument in the wavenumber range of 4000–400 cm^−1^. Some of ^1^H and ^13^C NMR data were measured with a Bruker Avance spectrometer (Bruker, Germany) at 400 and 100 MHz and others were measured with a Jeol spectrometer (Japan) at 500 and 125 MHz, respectively, using TMS as the internal standard. TMS was used as the internal standard and hydrogen coupling patterns were described as singlet (s), doublet (d), triplet (t), quartet (q) and multiplet (m). Chemical shifts were defined as parts per million (ppm) relative to the solvent peak. Antimicrobial activity was performed at Department of microbiology, Faculty of Pharmacy (Boys), Al-Azhar University, Cairo 11754, Egypt.

### General method for the synthesis of compounds 3 and 4

A mixture of thiosemicarbazone **1** (0.01 mol) and hydrazonoyl halides **2a, b** (0.01 mol) in ethanol containing trimethylamine was refluxed for 2 h. The colored precipitated solid was filtered on hot and recrystallized from DMF afforded the desired compounds **3** and** 4**.

### 6-Bromo-3-{1-[(4-phenyl-5-phenylazo-thiazol-2-yl)-hydrazono]-ethyl}-chromen-2-one (3)

Orange powder, yield: 80%; m.p: 235–236 °C; IR (KBr) cm^−1^: 3429 (N–H), 3068, 2924 (CH), 1735 (C=O), 1643 (C=N), 1599 (C=C); ^1^H NMR: (400 MHz, DMSO-*d*_6_, δ, ppm): 2.37 (s, 3H), 7.41 (t, 2H,* J* = 8.8 Hz), 7.58–7.64 (m, 5H), 7.73 (t, 1H, *J* = 8 Hz), 7.79, 7.81 (dd, 1H, *J* = 1.6 Hz, *J* = 2.4 Hz), 8.02 (d, 2H, *J* = 9.2 Hz), 8.23–8.26 (m, 3H), 8.30 (s, NH); ^13^C NMR (125 MHz, DMSO-*d*_6_ δ, ppm): 17.90 (CH_3_), 116.82, 118.75, 121.26, 123.05, 123.39, 127.37, 128.03, 129.27, 129.71, 130.78, 131.95, 134.61, 134.80, 135.35, 139.21, 140.94, 150.35, 151.79, 153.09, 158.93, 159.43 (Ar–C), 183.16 (C=O). Anal. calcd. for C_26_H_18_BrN_5_O_2_S (544.42): C, 57.36; H, 3.33; Br, 14.68; N, 12.86; S, 5.89. Found: C, 57.43; H, 3.26; Br, 14.75; N, 12.80; S, 5.97.

### (E)-2-(1-(6-bromo-2-oxo-2*H*-chromen-3-yl)ethylidene)hydrazine-1-carbimidic (Z)-2-ethoxy-2-oxo-N-phenylacetohydrazonic thioanhydride (4)

Yellow powder, yield: 77%; m.p: 209–210 °C; IR (KBr) cm^−1^: Broad 3428 (2N–H), 3050, 2921 (CH), 1736 (2C=O), 1599 (C=N), 1546 (C=C); ^1^H NMR: (400 MHz, DMSO-*d*_6_, δ, ppm): 1.31 (t, 3H,* J* = 7.6 Hz), 2.34 (s, 3H), 4.37 (q, 2H, *J* = 7.2 Hz), 7.23 (s, NH), 7.40–7.45 (m, 3H), 7.56 (t, 2H, *J* = 8.4 Hz), 7.79, 7.83 (dd, 2H, *J* = 8.8 Hz, *J* = 8.8 Hz), 7.95 (d, 1H, *J* = 8.9 Hz), 8.19 (s, 1H), 8.26 (s, NH), 8.59 (s, NH); ^13^C NMR (125 MHz, DMSO-*d*_6_ δ, ppm): 14.31, 17.89 (2CH_3_), 63.40 (CH_2_), 118.76, 118.94, 122.54, 122.85, 127.95, 129.08, 129.68, 131.93, 135.33, 139.12, 140.90, 141.17, 153.08, 153.48, 158.47, 159.11, 166.28 (Ar–C), 195.45 (2C=O). Anal. calcd. for C_22_H_20_BrN_5_O_4_S (530.39) C, 49.82; H, 3.80; Br, 15.07; N, 13.20; S, 6.05 Found: C, 49.78; H, 2.87; Br, 15.17; N, 13.11; S, 60.08.

### General method for the synthesis of compounds 6–9

A mixture of enaminone **5** (0.01 mol) and each of ethyl acetoacetate, acetylacetone, trifloroacetylacetone or ethyl cyanoacetate (0.01 mol) in acetic acid containing ammonium acetate was heated under reflux for 2 h. The colored solid obtained on hot was filtered, washed with ethanol, dried and recrystallized from DMF/EtOH mixture yielding the title compounds **6–9**.

### 6-(6-Bromo-2-oxo-2*H*-chromen-3-yl)-2-methyl-nicotinic acid ethyl ester (6)

Beige crystals, yield: 78%; m.p: 220–222 °C; IR (KBr) cm^−1^: 3059, 2986 (CH), 1740, 1715 (2C=O), 1612 (C=N), 1563 (C=C); ^1^H NMR: (400 MHz, DMSO-*d*_6_, δ, ppm): 1.34 (t, 3H, *J* = 7.2 Hz), 2.90 (s, 3H), 4.29 (q, 2H, *J* = 6.8 Hz), 7.28 (d, 1H, *J* = 8.8 Hz), 7.66, 7.68 (dd, 1H, *J* = 2 Hz, *J* = 2 Hz), 7.93 (s, 1H), 7.97 (d, 1H, *J* = 2 Hz), 8.19–8.24 (m, 1H), 8.85 (s, 1H); ^13^C NMR (100 MHz, DMSO-*d*_6_ δ, ppm): 14.53, 25.06 (2CH_3_), 61.62 (CH_2_), 116.83, 118.68, 121.38, 125.73, 132.13, 135.67, 139.44, 142.73, 152.74, 153.02 (Ar–C), 158.76, 166.21 (2C = O). Anal. calcd. for C_18_H_14_NBrNO_4_ (388.21) C, 55.69; H, 3.63; Br, 20.58; N, 3.61. Found: C, 55.60; H, 3.75; Br, 20.66; N, 3.69.

### 3-(5-Acetyl-6-methyl-pyridin-2-yl)-6-bromo-chromen-2-one (7)

Beige powder, yield: 82%; m.p: 230–233 °C; IR (KBr) cm^−1^: 3070, 2987 (CH) 1728, 1681 (2C=O), 1613 (C=N), 1562 (C=C); ^1^H NMR: (400 MHz, DMSO-*d*_6_, δ, ppm): 2.62 (s, 3H), 2.71 (s, 3H), 6.8 (d, 1H, *J* = 8.4 Hz), 7.39–7.53 (m, 1H), 7.79 (t, 1H, *J* = 9.2 Hz), 8.22–8.51 (m, 2H), 8.88 (s, 1H); ^13^C NMR (100 MHz, DMSO-*d*_6_ δ, ppm): 25.03, 29.97 (2CH_3_), 116.83, 118.68, 119.93, 121.32, 125.34, 127.02, 132.09, 135.35, 138.76, 142.49, 152.96, 157.39, 159.38 (Ar–C), 168.38, 170.41 (2C=O). Anal. calcd. for C_17_H_12_BrNO_3_ (358.19) C, 57.00; H, 3.38; Br, 22.31; N, 3.91. Found: C, 57.08; H, 3.29; Br, 22.41; N, 3.99.

### 3-(5-Acetyl-6-trifluoromethyl-pyridin-2-yl)-6-bromo-chromen-2-one (8)

Beige Powder, yield: 80%; m.p: 300 °C; IR (KBr) cm^−1^: 3066, 2922 (CH), 1731, 1682 (2C=O), 1645 (C=N), 1562 (C=C); ^1^H NMR: (300 MHz, DMSO-*d*_6_, δ, ppm): 2.43 (s, 3H), 6.86 (d, 1H, *J* = 6 Hz), 7.38 (d, 2H, *J* = 9 Hz), 7.79 (d, 1H, *J* = 8.7 Hz), 8.40 (d, 2H, *J* = 6 Hz). Anal. calcd. for C_17_H_9_BrF_3_NO_3_ (412.16) C, 49.54; H, 2.20; Br, 19.39; F, 13.83; N, 3.40. Found: C, 49.63; H, 2.29; Br, 19.49; F, 13.75; N, 3.47.

### 6-(6-Bromo-2-oxo-2*H*-chromen-3-yl)-2-oxo-1,2-dihydro-pyridine-3-carbonitrile (9)

 Yellow powder, yield: 75%; m.p: above 300 °C; ^1^H NMR: (400 MHz, DMSO-*d*_6_, δ, ppm): 6.9 (d, 1H, *J* = 4.4 Hz), 7.39–7.49 (m, 1H), 7.81, 7.83 (dd, 2H, *J* = 2 Hz, J = 2.4 Hz), 7.95 (s, NH), 8.49 (s, 2H). Anal. calcd. for C_15_H_7_BrN_2_O_3_ (343.13) C, 52.50; H, 2.06; Br, 23.29; N, 8.16. Found: C, 52.58; H, 2.13; Br, 23.20; N, 8.23.

### General method for the synthesis of pyrazoles 10a–c

To a mixture of the appropriate hydrazonoyl halide **2b–d** (0.01 mol) and the enaminone **5** (0.01 mol) in dry benzene (10 ml), was added triethylamine (0.2 ml) and the mixture was refluxed for 2 h. The mixture was filtered on hot and let to cool, the precipitate obtained after cooling was washed with EtOH, dried and recrystallized from DMF/EtOH mixture.

### 4-(6-Bromo-2-oxo-2*H*-chromene-3-carbonyl)-1-phenyl-1*H*-pyrazole-3-carboxylic acid ethyl ester (10a)

Orange powder, yield: 80%; m.p: 150–152 °C IR (KBr) cm^−1^: 3137, 3060 (CH), 1745, 1720, 1645 (3C=O), 1603 (C=N), 1556 (C=C); ^1^H NMR: (400 MHz, DMSO-*d*_6_, δ, ppm): 1.16 (t, 3H, *J* = 6.8 Hz), 4.20 (q, 2H, *J* = 6.8 Hz), 7.36–8.09 (m, 7H), 8.17 (s, 1H), 8.24 (s, 1H), 8.56 (s, 1H). Anal. calcd. for C_22_H_15_BrN_2_O_5_ (467.27) C, 56.55; H, 3.24; Br, 17.10; N, 6.00. Found: C, 56.44; H, 3.32; Br, 17.19; N, 6.11.

### 3-(3-Acetyl-1-phenyl-1*H*-pyrazole-4-carbonyl)-6-bromo-chromen-2-one (10b)

Yellow crystal, yield: 82%; m.p: 232–233 °C IR (KBr) cm^−1^: 3248, 3151 (CH), 1739, 1681, 1634 (3C=O), 1603 (C=N), 1554 (C=C); ^1^H NMR: (500 MHz, DMSO-*d*_6_, δ, ppm): 2.54 (s, 3H), 7.44–7.57 (m, 5H), 7.87–8.16 (m, 3H), 8.48 (s, 1H), 9.20 (s, 1H); ^13^C NMR (125 MHz, DMSO-*d*_6_ δ, ppm): 27.83 (CH_3_), 116.99, 119.09, 120.03, 120.80, 123.96, 128.18, 128.76, 130.35, 132.71, 134.30, 136.74, 138.86, 144.45, 149.95, 153.74, 158.36 (Ar–C), 184.74, 194.51 (3C=O). Anal. calcd. for C_21_H_13_BrN_2_O_4_ (437.24) C, 57.69; H, 3.00; Br, 18.27; N, 6.41. Found: C, 57.77; H, 2.91; Br, 18.35; N, 6.49.

### 4-(6-Bromo-2-oxo-2*H*-chromene-3-carbonyl)-1-(4-nitro-phenyl)-1*H*-pyrazole-3-carboxylic acid ethyl ester (10c)

Beige powder, yield: 75%; m.p: 220–222 °C ^1^H NMR: (500 MHz, DMSO-*d*_6_, δ, ppm):1.35 (t, 3H, *J* = 6.65 Hz), 4.44 (q, 2H, *J* = 6.7 Hz), 7.33 (s, 1H), 7.48 (d, 1H, *J* = 8.55 Hz), 7.64–7.72 (m, 5H), 8.55 (s, 1H), 9.15 (s, 1H); ^13^C NMR (125 MHz, DMSO-*d*_6_ δ, ppm): 14.61 (CH_3_), 62.42 (CH_2_), 117.00, 117.28, 119.68, 124.28, 127.02, 128.09, 128.50, 129.37, 133.26, 134.99, 137.52, 138.63, 150.80, 151.80 (Ar–C), 161.98 (3C=O). Anal. calcd. for C_22_H_14_BrN_3_O_7_ (512.27) C, 51.58; H, 2.75; Br, 15.60; N, 8.20. Found: C, 51.49; H, 2.80; Br, 15.66; N, 8.12.

### Synthesis of 5-[3-(6-bromo-2-oxo-2*H*-chromen-3-yl)-3-oxo-propenylamino]-1-phenyl-1*H*-pyrazole-4-carboxylic acid ethyl ester (12)

A mixture of enaminone **5** (0.01 mol) and 5-Amino-1-phenyl-1*H*-pyrazole-4-carboxylic acid ethyl ester (**11**) (0.01 mol) in 10 ml ethanol and few drops of acetic acid was heated in reflux for 2 h. The solid that is separated on hot was filtered, dried and recrystallized from DMF. Brown powder, yield: 79%; m.p: 120–121 °C; IR (KBr) cm^−1^: 3396 (N–H), 3074, 2989, 2944 (CH), 1730, 1682 (3C=O), 1622 (C=N), 1556 (C=C); ^1^H NMR: (400 MHz, DMSO-*d*_*6*_, δ, ppm): 1.26 (t, 3H, *J* = 6.8 Hz), 4.19 (q, 2H, *J* = 6.8 Hz), 6.33 (s, 2H), 7.38–8.21 (m, 10H), 8.59 (s, NH); ^13^C NMR (100 MHz, DMSO-*d*_6_ δ, ppm): 14.93 (CH_3_), 59.45 (CH_2_), 95.22, 116.54, 116.82, 118.59, 118.81, 120.47, 121.15, 124.05, 127.97, 129.91, 132.97, 137.01, 138.33, 140.62, 146.10, 150.19, 153.32 (Ar–C), 154.06, 158.76, 164.03 (3C=O). Anal. calcd. for C_24_H_18_BrN_3_O_5_ (508.32): C, 56.71; H, 3.57; Br, 15.72; N, 8.27. Found: C, 56.80; H, 3.66; Br, 15.82; N, 8.36.

### Synthesis of 5-(6-bromo-2-oxo-2*H*-chromen-3-yl)-2-thioxo-2,3-dihydro-1*H*-pyrido[2,3-*d*]pyrimidin-4-one (14)

A mixture of enaminone **5** (0.01 mol) and 6-aminothiouracil 14 (0.01 mol) in glacial acetic acid was heated in reflux for 2 h. The solid that is separated on hot was filtered, dried and recrystallized from DMF. Orange powder; yield: 77%; m.p: above 300 °C; IR (KBr) cm^−1^: 3425, 3320 (2N-H), 3072, 2902 (CH), 1733, 1675 (2C=O), 1610 (C=N), 1557 (C=C), 1172 (C=S); ^1^H NMR: (500 MHz, DMSO-*d*_6_, δ, ppm): 7.41 (d, 1H, *J* = 8.55 Hz), 7. 80 (s, 1H), 8.03–8.09 (m 2H), 8.34, 8.95 (2 s, 2H), 12.56, 13.11 ppm (s, 2NH); ^13^C NMR (125 MHz, DMSO-*d*_6_ δ, ppm): 112.38, 117.05, 118.93, 120.56, 121.10, 125.16, 131.92, 136.18, 137.73, 143.60, 151.67, 153.23, 156.00, 159.01 (Ar–C), 162.83, 176.6 (2C=O). Anal. calcd. for C_16_H_8_BrN_3_O_3_S (402.22) C, 47.78; H, 2.00; Br, 19.87; N, 10.45; S, 7.97. Found: C, 47.72; H, 2.06; Br, 19.81; N, 10.49; S, 7.94.

## Biological studies

### Antimicrobial activity

Evaluation of the antimicrobial activity of the prepared compounds was performed against four bacteria and two fungal species using the agar plate diffusion method^57^. Penicillin G and ciprofloxacin were used as reference drugs for gram positive and gram negative bacteria, while ketoconazole was used as standard antifungal drug.

### MIC Measurement

The microdilution method (broth dilution)^58^ was performed to measure the minimum inhibitory concentration (MIC) and minimum bactericidal concentration (MBC). Two fold serial dilutions of the test compounds (up to 10) and one quality control (QC) antibiotic in a microdilution plate. (Start with 1000 µg, 500 µg, 250 µg, 125 µg, 62.5 µg, 31.3 µg, 7.81 µg, 3.91 µg and 1.95 µg/ml). Create the inoculum by taking a few colonies from an agar plate with a sterile swab, prepare overnight broth then from broth preparing a McFarland standard (half McFarland ), and diluting the McFarland standard into media. (With Optical Density 0.1 at wavelength 580 nm). Dispense the inoculum into the microdilution plate with the serial diluted test compounds and incubate the microdilution plate overnight. Read the microdilution plate to determine the MIC value. Plate a portion of each well on an appropriate agar media, incubate the agar, and check for colonies to determine the MBC and MFC. The MIC was defined as the lowest concentration of the compound at which no visible growth occurred after 48 h of inoculation. The MBC showing the lowest concentration at which no visible growth occurred after 96 h of inoculation.

## Conclusions

This study describes the activity of new coumarin-based thiazoles, pyridines and pyrazoles as antimicrobial agents. The newly synthesized compounds were screened for in vitro antimicrobial activity against two gram + bacteria, two gram – bacteria as well as two fungal strains. Compound **12** was found to be the most potent compound against *B. pumilis* with MIC of 7.69 (µmol/ml). Compounds **3**,** 4** and **12** showed remarkable activity against *S. faecalis* with MIC of 14.34, 3.67 and 15.36 (µmol/ml), respectively. Regarding *E. coli*, most compounds recorded high to moderate MIC values (4.73–45.46 µmol/ml). Moreover, in case of *E. cloacae* compound **9** was the most potent compound with MIC value of 22.76 (µmol/ml).

## Data Availability

All data generated or analyzed during this study are included in this published article and its supplementary information file.
